# Plasma-derived exosomal miR-326, a prognostic biomarker and novel candidate for treatment of drug resistant pediatric acute lymphoblastic leukemia

**DOI:** 10.1038/s41598-023-50628-w

**Published:** 2024-01-06

**Authors:** Neda Saffari, Soheila Rahgozar, Elaheh Faraji, Fikrettin Sahin

**Affiliations:** 1 Department of Cell and Molecular Biology and Microbiology, Faculty of Biological Science and Technology, University of Isfahan, Hezar jarib Street, Isfahan, 81746-73441 Iran; 2 Department of Genetics and Bioengineering, Yeditepe University, Atasehir, 34755 Istanbul, Turkey

**Keywords:** Cancer, Cell biology, Molecular biology, Biomarkers, Medical research, Molecular medicine, Oncology

## Abstract

Acute lymphoblastic leukemia (ALL) is a cancer with high incidence rate in pediatrics and drug resistance is a major clinical concern for ALL treatment. The current study was designed to evaluate the role of exosomal miR-326 in diagnosis and treatment of children with B-ALL. Exosomes were isolated from plasma samples of 30 patients and B-ALL cell lines followed by characterization, using nanoparticle tracking analysis, immunoblotting assay and electron microscopy. qPCR showed significantly increased levels of miR-326 in patients exosomes compared with non-cancer controls (*P* < 0.05, AUC = 0.7500). Moreover, a comparison between the sensitive and drug resistant patients revealed a prognostic value for the exosomal miR326 (*P* < 0.05, AUC = 0.7755). Co-culture studies on drug resistant patient primary cells and B-ALL cell lines suggested that exosomes with high miR-326 level act as vehicles for reducing cells viability. B-ALL cell line transfection with naked miR-326 mimic confirmed the results, and fluorescence microscopy validated uptake and internalization of exosomes by target cells. The novel introduced features of the exosomal miR-326 address a non-invasive way of diagnosing primary drug resistance in pediatric ALL and advocates a novel therapeutic strategy for this cancer.

## Introduction

Pediatric acute lymphoblastic leukemia (pALL) is the most prevalent cancer in children ^1^, with B-cell ALL being the predominant subtype. This malignancy arises from the uncontrolled proliferation of immature B-lymphocytes in the bone marrow, subsequently spreading to the blood and extramedullary sites^1,2^. Despite notable progress in recent years, the enduring survival rate for children who experience an initial relapse of ALL remains at a mere 50%. Moreover, subsequent relapses exhibit even more dismal outcomes^3^. The presence of approximately 30% lymphoblasts in bone marrow is considered as the diagnostic hallmark of ALL^2^. Moreover, identification of cells morphology, flow cytometry, immunophenotyping and cytogenetic tests are required for validation of the diagnosis and risk stratification^4,5^. The identification of novel diagnostic and prognostic biomarkers for ALL holds great promise in enabling timely detection of the disease, thereby enhancing patient outcomes, and mitigating the risk of relapse. Furthermore, it is important to note that current clinical tests possess certain limitations in accurately detecting thresholds of sensitivity and specificity, as well as involving bothersome procedures. However, the advent of a new class of biomarkers hold promise in addressing these challenges^6^.

Exosomes, the most valuable subset of extracellular vesicles (EVs), are stable nano-metered lipid bilayer-enclosed particles (30–200 nm) containing functional biomolecules including proteins, lipids, RNAs, and DNAs^7^. Exosomes could be considered as a homogenous population of extra-cellular vesicles (EVs), specially in an imposed condition^8–11^. Exosomes may be constitutively secreted by different types of cells, however, their content reflects the biological status of their parental cells^12,13^. Recent studies have noted the importance of exosomes in physiological and pathological intercellular communications^14–16^. These nanovesicles, initially considered as trash bins, have now emerged as gold mines which are exported by cells aiming different purposes. In normal cells, it seems that the purpose of nanovesicles exocytosis is removing of extra biomolecules from the cell. However, in the context of cancer progression, this mechanism may be attributed to different goals. Numerous studies have confirmed that upon the nature of their contents, exosomes play important roles in proliferation, activating or inhibiting specific signaling pathways, promoting angiogenesis and metastasis, modulating immune system, and remodeling the surrounding microenvironment^17–19^. On the other hand, exosomes may be clinically considered as appropriate illustrators of ongoing procedures inside the cells which can be detected more earlier and in a convenient way.

MicroRNAs (miRNAs) are non-coding, small size RNAs (19–25 nucleotides) which may be selectively exported into exosomes^20,21^. MiRNAs can act as tumor suppressor or oncogene and are involved in some cellular procedures such as cell growth, proliferation, differentiation and apoptosis^22^. miR-326 is introduced as a tumor suppressor^23–25^ which may interfere with Wnt/β-catenin pathway inducing drug resistance in Glioma and lung cancer^26,27^ or participate in drug exporting by targeting ABCA2/ABCC1 transporter in breast cancer^28,29^. Other candidate targets for miR-326 are transcription factor 4^30,31^ and Bcl-2^32,33^, through which it may prevent cell proliferation in endometrial cancer malignant cells. Interestingly, a negative correlation between the expression levels of miR-326 and Bcl-2 was previously shown in pediatric B-ALL^35^. Moreover, our previous investigations elucidated the poor prognostic role of the decreased levels of this non-coding RNA in pediatric ALL patients^20,28,34,35^. The aim of this study was to analyze the differential expression of exosomal miR-326 compared to the control group in children with pALL and to determine the effect of this exosome-transported miroRNA on target cells.

## Materials and methods

### Cell culture

A CD10^−^/CD34^−^, RN95 cell line, was established, in-house, from a multiple relapsed pediatric B-ALL patient^36^ (national patent number: 100281). Nalm6 cell line was purchased from the cell bank of Pasteur Institute in Iran. Cells were grown in RPMI1640 (Bio Idea, Tehran, Iran) containing 1% penicillin/streptomycin and 1% L-glutamine. Culture media were supplemented with 20% and 10% heat-inactivated FBS (Bio Idea, Tehran, Iran) for Nalm6 and RN95, respectively. cells were then incubated at 37 °C in a humidified atmosphere of 5% CO_2_.

### Patients and sampling

Sampling was achieved with full written informed parent’s consent. Peripheral blood was obtained from children with acute lymphoblastic leukemia who referred to the Sayed-ol-Shohada Hospital (Isfahan, Iran) from 2021 to 2022 February first, and age matched healthy donors. The inclusion criteria for patients were the diagnosis of pre-B-ALL at the age between 1 and 14 years, confirmed by flow cytometry and the presence of over 30% blast in the bone marrow aspirate. The exclusion criteria were diagnosis of Burkit lymphoma, identification of Philadelphia chromosome translocation t(9:22) (q34;q11) and impossibility for one year follow-up due to patient non-attendance. 3 patients and healthy donors entered the screening phase of the study. The number of patients and controls in validation phase was 30 and 10, respectively^37,38^. The study was conducted in accordance with the Declaration of Helsinki, and permitted by the Ethics Committee of the University of Isfahan (agreement number: IR.UI.REC.1398.010). Response to treatment in the ALL patients was assessed using the presence of minimal residual disease (mrd), a year after treatment. Samples were transferred to the University on ice for preanalytical procedures. Plasmas were isolated from the whole blood and stored at  − 70 °C freezer.

### Exosome extraction

#### Patient samples

10 ml peripheral blood was obtained from the cases involved in the screening phase. In order to harvest platelet-free plasma (PFP), samples were centrifugated twice at 2500 g for 15 min at RT. PFP was diluted in equal volume with PBS and then centrifugated at 12,000 g for 45 min at 4 °C. Then, supernatant was harvested and filtered through 0.22 µm filters (Millipore, Burlington, MA). Filtered supernatant was subjected to ultracentrifugation twice at 110,000 g for 2 h using an ultracentrifuge (Beckman Coulter, Brea, CA). The supernatant was removed and the pellet was resuspended in PBS. The tube was filled with PBS and subjected to ultracentrifugation for 70 min at 110,000 g. Exosomes were pelleted at the final centrifugation step and were resuspended in 1ml PBS. The extracted exosomes were filtered using 0.22 µm filter and stored at  − 80 °C in 200 µl aliquots.

500 ul plasma was used as starting material for isolating exosomes from each sample incorporated in the validation phase. Exosomes were isolated using ExoQuick™ (System Biosciences, Palo Alto, CA) according to the manufacturer protocol with minor modification. Briefly, samples were centrifuged at 3000 g for 15 min. For each sample, the Supernatant was transferred to a sterile vessel and filtered using 0.22 syringe filter. 63 µl ExoQuick solution was added to 500 ul clarified plasma. Samples were inverted completely and then incubated at 4°C overnight. The mixture was centrifuged at 1500 g for 30 and 5 min, respectively, at RT in order to pellet the exosomes. The pellet was then resuspended in 200 μl of PBS for downstream analysis^39,40^.

#### Culture media

In the screening phase, exosomes of Nalm6 and RN95 cell lines were isolated by differential ultracentrifugation according to the protocol described by Thery et al.^41^. 40 ml conditioned media were used as biological fluids for extracting exosomes. Each cell line was grown in regular complete media to a high-density cell population in logarithmic phase. Then, regular complete media was substituted thoroughly with FBS-free complete media for exosome production. After 24h Nalm6/RN95, and 48h RN95 cultivation in exosome production media, supernatant was harvested and subjected to several steps of ultracentrifugation using a fixed angle rotor centrifuge (Gyrozen, Daejeon, Korea). The supernatant was initially centrifuged at 300 g for 10 min, followed by 10,000 g for 30 min at 4 °C. Subsequently, the cleared supernatant underwent centrifugation at 100,000 g for 70 min with swinging-bucket rotors (Beckman coulter, Brea, CA). In the subsequent washing step, EVs were washed with PBS and the final EV particles were resuspended in 1 ml PBS. Followed by filtering of EV particles, using 0.22 µm filter. Aliquots of exosomes were stored at 80°C for further analysis.

### Characterization of exosomes

#### NTA

Concentration and size distribution of isolated exosomes from plasma and conditioned media were detected using Nanosight NS300 particle counter (Malvern Panalytical, Malvern, UK)^42,43^. The system was equipped with the LM14 laser module with a 488 nm blue laser. All samples were diluted in order to obtain 20–100 particles in the frame (10^7^–10^9^ particles/ml). Laser module was removed from the device and 1 ml diluted sample was injected into the module. Five video captures were recorded by sCMOS camera for all samples with 60 s intervals. Camera level was selected on 16 for analyzing video captures. appropriate setting and detection threshold were applied to the measurements. NTA software version 3.4 build 3.4.003 was used for setting and analyzing the results.

#### BCA assay

Before the quantification of total protein concentration, exosomes were resuspended in PBS and cell suspensions were lysed using RIPA lysis buffer system (Santa Cruz, Dallas, TX). Equal volume of RIPA buffer containing PMSF, protease inhibitor cocktail and sodium orthovanadate in water was added to every aliquot. Calculation of total protein concentration of different aliquots were assessed using a Pierce™ BCA Protein Assay Kit (Thermo Fisher Scientific, Waltham, MA) according to the manufacturer protocol. Results were measured using a plate reader (Bio Tek, Winooski, VT).

#### Traditional and capillary automated western blotting

In traditional immunoblotting, extracellular vesicles and cell suspensions were examined using different anti-human(αh)primary antibodies including rabbit-αhGM130(1:400), Hsp70(1:200), Flotillin-1 (1:200) and CD9 (1:200) (All purchased from Cell signaling, Danvers, MA), in addition to mouse-αhCD63 (Thermo Fisher Scientific, Waltham, MA)^42,44,45^. Secondary antibodies were goat anti-rabbit and horse anti-mouse antibodies (1:5000) (Cell signaling, Danvers, MA). 1 to 8 µg exosomal/cellular protein was employed for traditional immunoblotting. Extracellular vesicles and cell suspensions were resuspended in the loading buffer and boiled at 99°C for 5 min with or without β-mercaptoethanol. Protein of exosomes and cell aliquots were separated by 4–20% Gradient sodium dodecyl sulphate–polyacrylamide gel (4–20%) (Biorad, Hercules, CA) electrophoresis. Proteins were transferred onto PVDF membrane (Biorad, Hercules, CA) by Wet/Tank Blotting system (Biorad, Hercules, CA). Membranes were blocked with 5% skimmed milk in 1 × TBS/Tween solution for 1 h followed by incubation with primary antibodies for 1.5 h at RT and overnight at 4°C. After washing with 1 × TBS/Tween solution, membranes were incubated with secondary antibody. Membranes were visualized using ECL solutions (Immobilon, Temecula, CA) and the ChemiDoc system (Biorad, Hercules, CA). Image Lab Software 6.0.1 was used for quantification of protein bands.

In capillary immunoblotting^46–49^, mouse-αhCD81 (1:100) (Invitrogen, Waltham, MA), rabbit-αh Flotillin-1(1:25) and rabbit-αhHsp70 (1:50) (Cell Signaling, Danvers, MA) were used as primary antibodies. Anti-rabbit and anti-mouse Detection Modules (Protein Simple, San Jose, CA) were used for secondary antibody bindings. Capillary cartridges were filled with low concentration of proteins (0.02 to 0.2 µg/µl). Protein expression was shown by capillary Wes—automated Western blotting (Protein Simple, San Jose, CA). The calculation and analysis of protein expression were done according the gel-like images acquired by the Compass for SW software (Version 4.0, Protein Simple).

#### Field emission scanning and transmission electron microscopies (FE-SEM and FE-TEM)

The morphology of exosomes was analyzed using a field emission electron microscope (Brno, Czech Republic). Exosomes were resuspended and diluted in 1 ml of PBS and subjected to sample preparation according to the related protocols. Briefly, the copper holder was covered with glue and aluminum foil, then a drop of exosome sample was placed and dried in a vacuum machine. Subsequently, images were taken followed by gold sputterings. For TEM analysis, 400 mesh formvar coated copper grid and 2% uranyl acetate were used for negative staining. A drop of the diluted sample was placed on a formvar coated grid. After 2 min, the excess liquid was blot off with filter paper. A drop of 2%uranyl acetate solution was added and the excess liquid blot off with filter paper after 2 min. Negative stained sample was examined by 50 kV Transmission Electron Microscope, Zeiss EM 900 (Germany).

#### RNA extraction, cDNA synthesis and RT‑qPCR assay

Total exosomal RNAs were isolated using the Plasma/Serum RNA Purification Mini Kit (Norgen Biotek Corp, Thorold, Canada) according to the manufacturer protocol. The quality and quantity of the RNAs were measured by NanoDrop 1000 spectrophotometer (Thermo Fisher Scientific, Waltham, MA). The same amount of plasma samples was applied for exosomal miRNAs extraction. Complementary DNAs (cDNAs) were synthesized using microscript microRNA cDNA Synthesis Kit (Norgen Biotek Corp, Thorold, Canada) in Mastercycler thermocycler (Eppendorf, Hamburg, Germany). Real-time PCR was performed using SYBR Green 2X Real time PCR Kit (Norgen Biotek Corp, Thorold, Canada). For normalization of Ct values, the microRNA (cel-miR-39) Spike-In Kit (Norgen Biotek Corp, Thorold, Canada) was used as an exogenous control. All PCR reactions were done in duplicates during two separate experiments using Applied Biosystems StepOnePlus real-Time PCR system (Thermo Fisher scientific, Waltham, MA). Relative expression level of miRNA was normalized against that of cel-miR-39^50^ (for patient samples) or U6 (for cell lines) using the 2^−ΔCt^ method. The comparative 2^−∆∆CT^ method was used for data analysis and calculation of relative quantification of gene expression was performed using the following formula:

$$\Delta \Delta {\text{Ct}}\, = \,{\text{Patient }}\left( {{\text{Ct}}_{{{\text{miR}} - {326}}} - {\text{Ct}}_{{{\text{Cel}} - {\text{miR}} - {39}}} } \right){-}{\text{Control }}\left( {{\text{Ct}}_{{{\text{miR}} - {326}}} - {\text{Ct}}_{{{\text{Cel}} - {\text{miR}} - {39}}} } \right).$$

#### Exosome administration

RN95 cells were grown in T25 flask. The culture medium was RPMI-1640 supplemented with fetal bovine serum and 1% penicillin/streptomycin. The population of RN95 and Nalm6 cells were selected in high density for exosome isolation. Followed by 48 h FBS starvation, RN95 and Nalm6 exosomes were extracted from 44 ml conditioned media using ultracentrifugation method as mentioned earlier. The exosome pellet was resuspended in 1 ml PBS, then stored in  – 20℃ for next use. The concentration of surface proteins was determined using Bradford assay. In two separate experiments, RN95 and Nalm6 cells were cultured in 96-well tissue culture plates at a density of 10 × 10^4^ cells in 100 µl FBS-free media per well. Subsequently, 50 µl of RN95 and Nalm6 exosomes were added to the wells with different concentrations (1, ½, ¼). After 48 h incubation at 37℃, viability tests were performed.

#### Viability assays

Trypan blue assay was conducted by harvesting cells from the cultured media. Trypan blue dye was mixed with the cell suspension at a 1:1 ratio and % viability was calculated by counting the number of stained and unstained cells using hemocytometer. MTT assay was performed by adding10 μL of MTT solution (5 mg/mL) to each well of cultured cells. Following incubation at 37 °C for 3 h, 100 µl supernatant was removed and formazan crystals were solubilized by adding 100 μL DMSO. Absorbance was captured at 492 nm by using a stat fax-2100 microplate reader (Awareness Technology, Westport, CT).

#### Uptake analysis

After cell washing with PBS, 2 mg/ml DiI (CellTracker™ CM-DiI, Thermo Fisher Scientific, Waltham, MA) stock solution was prepared. Exosomes concentrations were assessed using lowry method. 20 µg/µl exosomes were labelled with 1mg/ml DiI and incubated in dark for 5 min at RT, then 20 min at 4℃. 100 µl labelled- exosomes were adjusted to RN95 cells in dark for 30 min at 4℃. Cell labelling was performed using 1 µg/ml DAPI (Sigma-Aldrich, Darmstadt, Germany). Cells were analyzed using fluorescence microscope (Labomed. Los Angeles, CA).

#### Treatment of patient primary cells with exosomes

Patient mononuclear primary cells were isolated using Ficoll–Hypaque (inno-train Diagnostik, Clinton, NY) density gradient centrifugation method, according to the manufacturer protocol. Peripheral mononuclear cells (MNCs) were cultured in 96-well tissue culture plates at a density of 10 × 10^4^ cells per well, in 100 µl FBS-free media. Subsequently, cells were treated with 50 µl of RPMI with and without (as control) exosomes. Following 48 h incubation, viability assays were performed. Relative gene expression level of exosomal miR-326 was quantified using 2^−ΔΔCt^ method.

#### Transfection of B-ALL cell line with miR-326 and collecting exosomes

RN95 cells were grown to a high density, then transferred to electroporation media. 250 nM miR-326 mimic and miRNA mimic control (scrambled) (Qiagen, Hilden, Germany) were delivered separately into RN95 cells (2 × 10^6^) using Multiporator electroporation system (Eppendorf, Hamburg, Germany). Another group of cells (mock control) were only subjected to electroporation device. Moreover, a fourth group of cells were selected as untreated control. Cells were electroporated 1 pulse (40 µS) at 250 voltage. After 48h incubation, cell viability assay was performed. cells were centrifuged at 200 g for 5 min and exosomes were extracted from 5 ml conditioned media using Exo-spin™ buffer (Cell Guidance Systems, Cambridge, UK) according to the manufacturer protocol for low-protein biological fluids. Cells were removed from the supernatant by centrifugation at 300 g for 10 min. Supernatant was transferred to a new centrifuge tube and spun at 16,000 g for 30 min to remove any remaining cell debris. Resulting supernatant was filtered through a 0.22 µm syringe filter. ½ volume of Exo-spin™ Buffer was added to the filtered supernatant and mixed well, then incubated overnight at 4°C. The mixture was centrifuged at 16,000 g for 1 h. Supernatant was aspirated carefully, and the exosome pellet was resuspended in 500 µl PBS. Subsequently, after extraction of exosomal miRNA using Exo-spin™ buffer (Cell Guidance Systems, Cambridge, UK), the exosomal miR326 expression level was measured using RT-qPCR.

### Statistical analysis

All data were reported as mean ± standard error of mean (SEM). *P* < 0.05 was considered as the significant difference. Data are results of 2 independent experiments in duplicates. For comparison between two groups, unpaired t or Mann–Whitney tests were performed. Ordinary one-way analysis of variance (One-Way ANOVA) was done for multiple group comparisons. GraphPad Prism 9.3.1 (GraphPad Software, San Diego, CA) software was used for analyzing results.

### Ethical approval

All the patients’ parents were informed about the purposes of the study and consequently have signed their consents. All investigations conformed to the principles outlined in the Declaration of Helsinki and were performed with permission by the responsible Ethics Committee of the University of Isfahan (Agreement number: IR.UI.REC.1398.010 for human studies).

## Results

### Isolation and characterization of the exosomes derived from acute lymphoblastic leukemia cell lines

**Figure 1:**
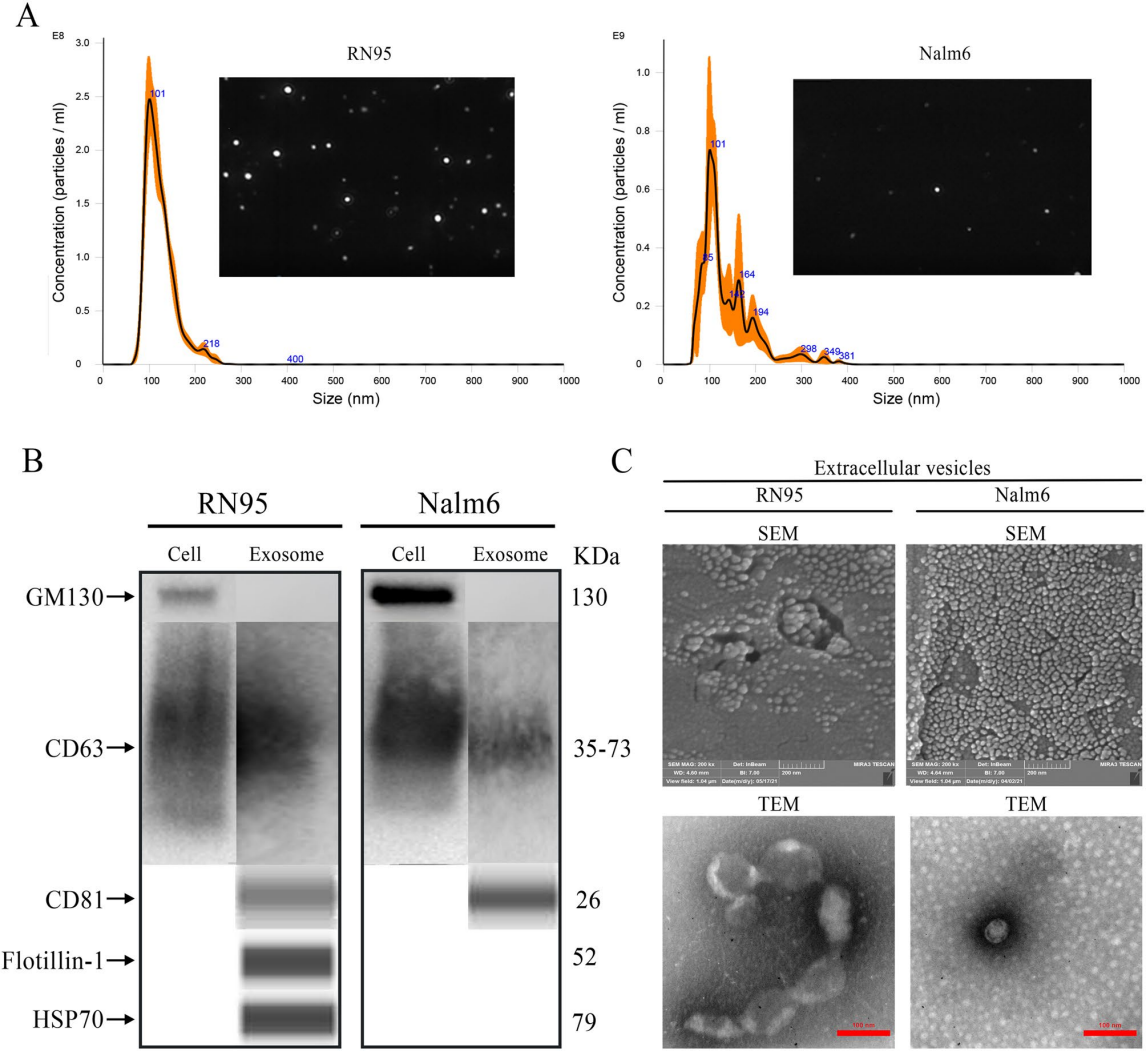
Isolation and characterization of the exosomes derived from acute lymphoblastic leukemia cell lines. (**A**) Representative histogram and a video image of particle concentration and size distribution of exosomes isolated from cell culture supernatant using Nanoparticle Tracking Analysis (NTA). Exosomes were extracted from the conditioned media of RN95 and Nalm6 cell lines using ultracentrifuge. The incubation time for RN95 and Nalm6 cells were 48h and 24h, respectively. The Orange error bar area indicates ± standard error of the mean, n = 5. (**B**) Confirmation of the presence of common exosomal markers on the extracted leukemia cell lines exosomes. Evaluation of CD63 (positive) marker and GM130 (negative) marker using semi quantitative traditional western blotting. Automated western blot analysis indicated the positive expression levels of CD81, FLOT1, and HSP70 in the isolated exosomes. CD63, Lysosome-associatd membrane protein-3; GM130, Cis-Golgi marker; CD81, Tetraspanin, non-specific transmembrane protein member 8; FLOT1, Flotillin-1; HSP70, Heat Shock Protein 70. (**C**) Field-emission scanning and transmission electron micrographs of the isolated exosomes from RN95 and Nalm6 conditioned media.

Nalm6 and RN95 cell lines were seeded in 6-well cell culture plates and exosomes were harvested from FBS-free media while cells viability was greater than 95% (Figure S1). Exosomes were diluted in PBS and subjected to nanoparticle tracking analyses. The videos that were recorded 5 times (every time 60 s) for each sample confirmed that no large-size or aggregated vesicle was extracted from the cells’ supernatant. The presence of exosomes was identified upon the viscosity and the Brownian motion of the nanoparticles. NTA data showed a high concentration of particles (1.44 × 10^10^) ± (9.58 × 10^8^) per milliliter with a size distribution of 123 nm and a mode size of 105 nm for RN exosomes. For the Nalm6 exosomes the size distribution was around 137 nm and their concentration was adequate for downstream analyses [(4.68 × 10^10^) ± (5.12 × 10^9^) particles/ml] (Fig. 1A). Evaluation of the quality of exosome isolation was performed by immunoblotting and using Wes™ Simple Western. Expression of the exosomal markers CD63^51–53^, CD81, FLOT1, HSP70, and the negative marker (GM130) was demonstrated. Therefore, the correct purification of exosomes without contamination from cellular components was confirmed (Fig. 1B). Isolated particles were assessed using FE-SEM and FE-TEM (Fig. 1C).

### Isolation and identification of exosomes from the B-ALL patient plasma using ultracentrifugation

**Figure 2:**
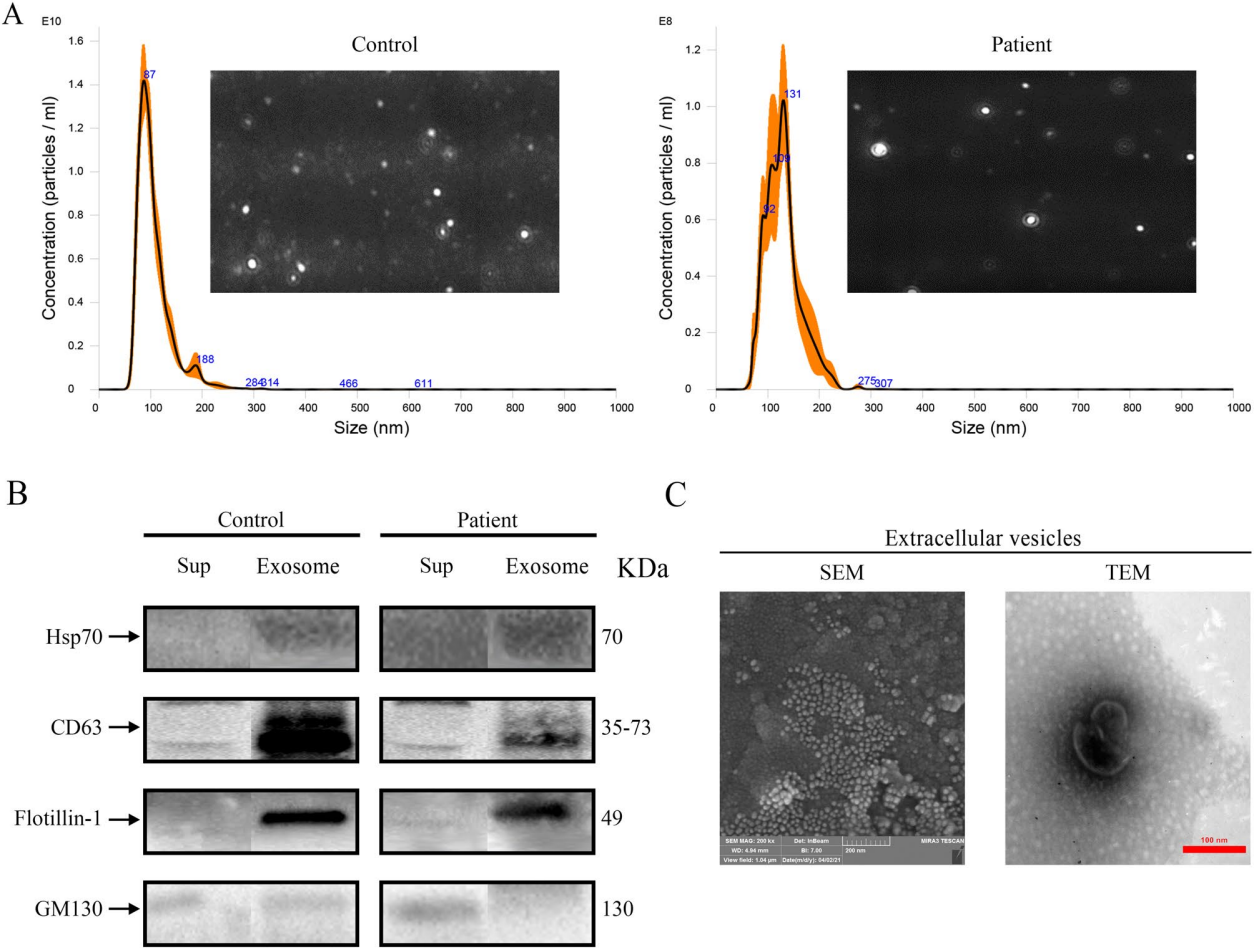
Isolation and characterization of the isolated exosomes from the plasma of children with acute lymphoblastic leukemia using ultracentrifugation as a gold standard method in comparison with control cases. (**A**) Representatives of a video image and the histogram using Nanoparticle Tracking Analysis demonstrating the size and concentration of exosomes isolated from the plasma of a patient and a healthy donor as control. The Orange error bar area indicates ± standard error of the mean, n = 5. (**B**) Validation of the presence of exosomal markers in the extracted exosomes from plasma. The presence of HSP70, CD63, FLOT1, and CD9 as positive exosomal proteins and GM130 as a negative marker have been shown using traditional western blotting; Sup, Supernatant; exosome, exosome pellet; HSP70, Heat Shock Protein 70; CD63, Lysosome-associated membrane protein-3; FLOT1, Flotillin-1; GM130, Cis-Golgi marker. (**C**) Field-emission Scanning Electron Micrograph and Transmission Electron Micrograph of exosomes isolated from ALL patient plasma using ultracentrifugation method.

Blood derived from a patient with B-ALL and a healthy donor as control was processed by differential centrifugation to separate platelets-free plasma (PFP) from blood cells. Exosome-enriched pellets were obtained from PFP using ultracentrifugation. Exosomes were, then, assessed by a tracking analyzer. The related data showed that the average size of the captured exosomes is below 200 nm for both patient and control samples. The mean and mode size of the exosome populations were 129 and 130 for the patient and 106 and 87 for the control samples, respectively. Moreover, the concentration of exosomes for the patient and the control sample were reported as (6.79 × 10^9^) ± (4.49 × 10^8^) versus (7.11 × 10^11^) ± (2.74 × 10^10^) particles/ml, respectively (Fig. 2A). The presence and absence of the established exosome markers were assessed in the exosome lysates and exosome-depleted supernatants, respectively. Lysates showed detectable HSP70, CD63, FLOT1, and CD9 as positive exosome markers. Moreover, GM130, a negative exosome marker, was not identified in any of the lysates. Results showed successful purification and characterization of exosomes from trace amounts of the patient plasma (Fig. 2B). Moreover, scanning and transmission electron microscopy confirmed the exosomal origin of the characterized particles. Exosomes showed spherical shape and a diameter of 30–200 nm (Fig. 2C). Furthermore, performance of the exosome isolation kit in purifying exosomes from plasma fluid was evaluated upon comparison of data with the ultracentrifugation method. The NTA, Western blot and electron microscopy data obtained from exosome samples using the commercial kits confirmed the reliability and efficacy of the isolation kits.

### Encapsulation of miR-326 transcript inside the culture media collected exosomes of B-ALL cell lines

**Figure 3:**
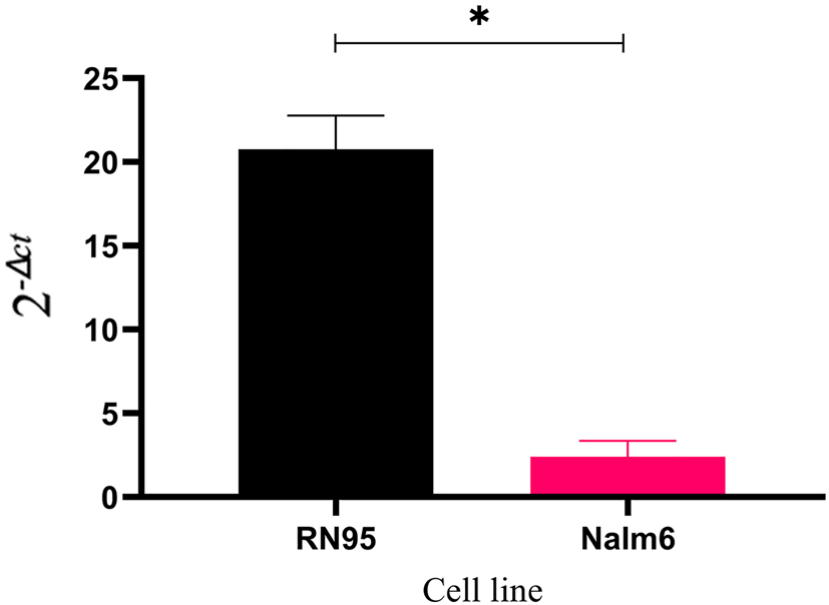
Expression of miR-326 transcript in exosomes isolated from the conditioned media of RN95 and B-ALL Nalm6 cell lines. Bar graph representing the expression level (2^−Δct^) of exosomal miR-326 transcript in the cell culture supernatant of RN95 and Nalm6 cell lines, respectively. ΔCt value is normalized against Cel-miR-39 as exogenous control. Exosomal miR-326 is expressed in two conditioned media with a high level in RN95 compared with Nalm6 media.

Exosomes were collected from the conditioned media of B-ALL cell lines (RN95 and Nalm6) and the exosomal expression levels of miR-326 transcript was examined using RT-qPCR method. Results showed that the exosomes released in the supernatant of RN95 cell line contain 10 times more amount of miR-326 transcript than the supernatant of Nalm6 cell line (Fig. 3).

### Expression level and diagnostic value of exosomal miR-326 in B-ALL patients samples

**Figure 4:**
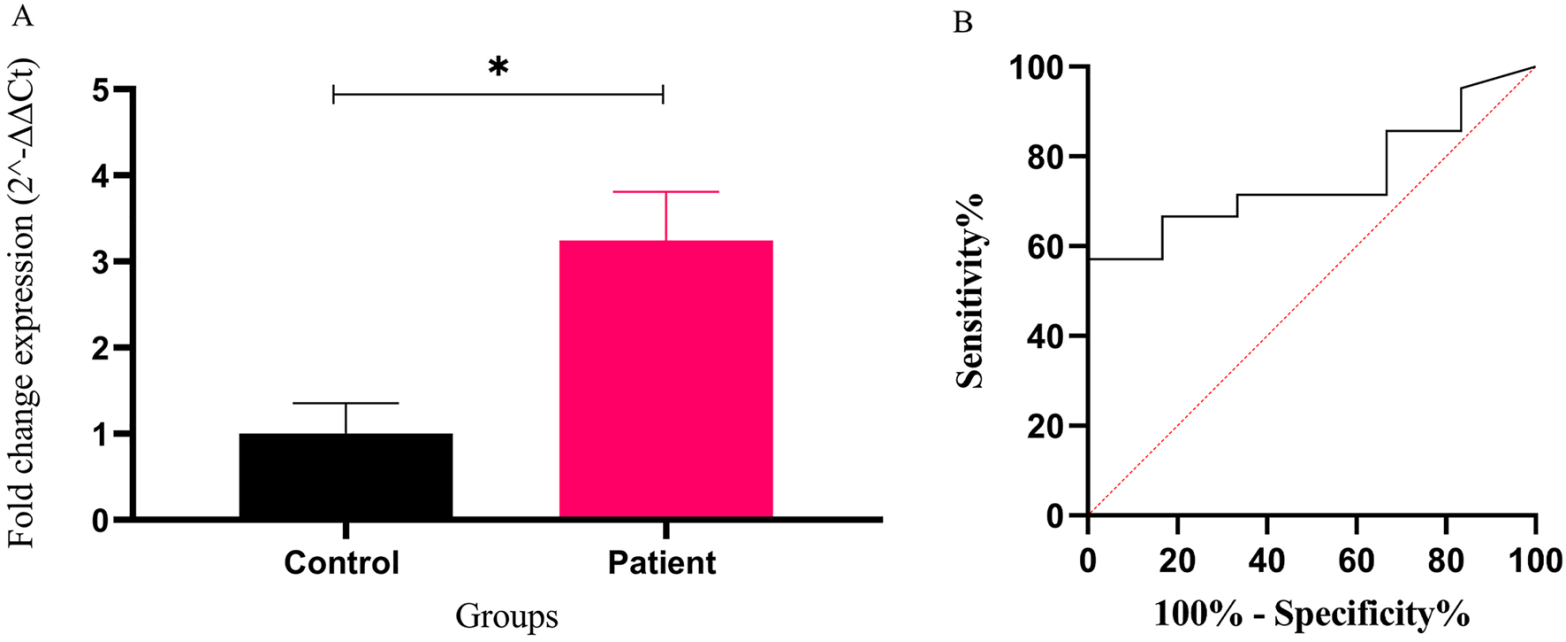
The diagnostic value of exosomal miR-326 expression in B-ALL patients samples. (**A**) the Relative expression of exosomal miR-326 was assessed in B-ALL patients and control cases using RT-qPCR. T-test analysis showed a significant difference between the expression levels of exosomal miR-326 in B-ALL subjects compared with the control group. (**B**) ROC curve analysis showed that the exosomal miR-326 expression levels could distinguish B-ALL cases from healthy controls. ROC, receiver operating characteristic; AUC, area under the ROC curve. Values are mean ± SEM in duplicates, *P value < 0.05.

The relative expression of exosomal mir-326 was compared between de novo B-ALL patients and control group. qPCR results determined that the expression profile of exosomal miR-326 in ALL patients was increased compared with the control group [0.58 ± 0.2% vs. 1.88 ± 0.32 (mean ± SEM), *P < 0.05] (Fig. 4A). ROC analysis suggested that the exosomal miR-326 expression level may be considered as a possible biomarker for distinguishing B-All patients from control cases. Area under the ROC curve value showed good performance of exosomal miR-326 expression level in discriminating between two groups (AUC = 0.7500, 95% confidence interval) with a sensitivity of 57.14% and specificity of 100% (Fig. 4B). Moreover, EVmiRNA database, confirmed that miR-326 has exosome loading capacity and blood is the right tissue for tracking exosomal miR-326 (Figure S2).

### Evaluation of the prognostic significance of the exosomal miR-326 expression level in pediatric B-ALL patients

**Figure 5:**
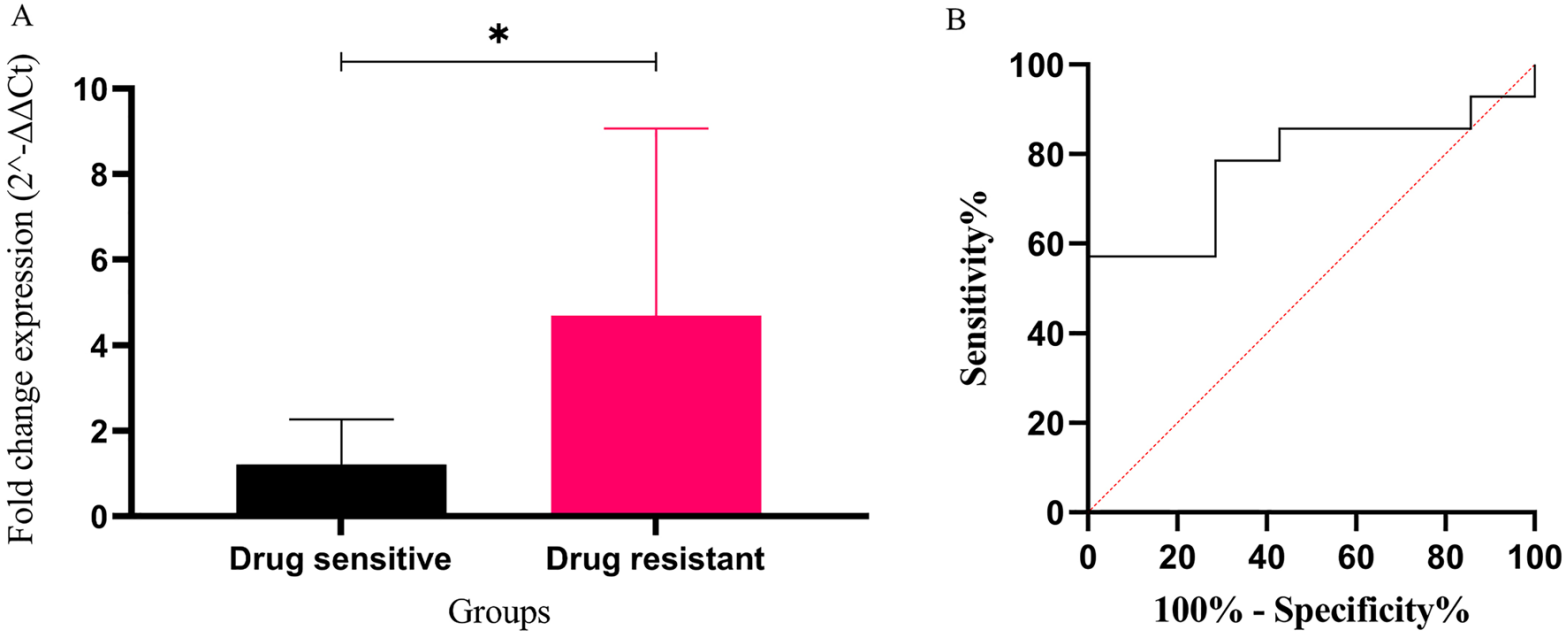
The prognostic effect of exosomal miR-326 expression in B-ALL patients samples. (**A**) Comparison between the mrd− and MDR groups was assessed according to the exosomal miR-326 expression levels using RT-qPCR. Significant upregulation of exosomal miR-326 expression was demonstrated in the MDR group. (**B**) ROC curve analysis showed significant prognostic potential for exosomal miR-326 expression level in discriminating MDR patients from the sensitive cases (AUC = 0.7755). MDR, multidrug-resistant (relapsed patients in addition to the mrd + patients, identified as those resistant to chemotherapy); mrd, minimal residual disease; mrd−, sensitive patients to chemotherapy with absence of post treatment minimal residual disease. Values are mean ± SEM in duplicates,*P < 0.05.

According to the outcome of response to chemotherapy after one year receiving chemotherapy, patients were classified into those with minimal residual disease (mrd+) and those who were drug sensitive with no presence of mrd in their bone marrow (mrd−). mrd+ and relapsed individuals were identified as resistant patients to chemotherapy. The exosomal miR-326 expression level was compared between sensitive and drug resistant groups using acquired data from RT-qPCR [1.213 ± 0.3% vs. 4.684 ± 1.1% (mean ± SEM), *P* < 0.05] (Fig. 5A). In order to identifying the possible prognostic value of exosomal miR-326 expression in discriminating resistant cases from sensitive patients ROC analysis was performed. The AUC index validated this prognostic significance of miR-326 with good performance (AUC = 0.7755, 95% confidence interval,) and a sensitivity of 57.14% and specificity of 100 (Fig. 5B).

### Reduced viability of exosome-treated B-ALL cell lines followed by exosomes uptake

**Figure 6:**
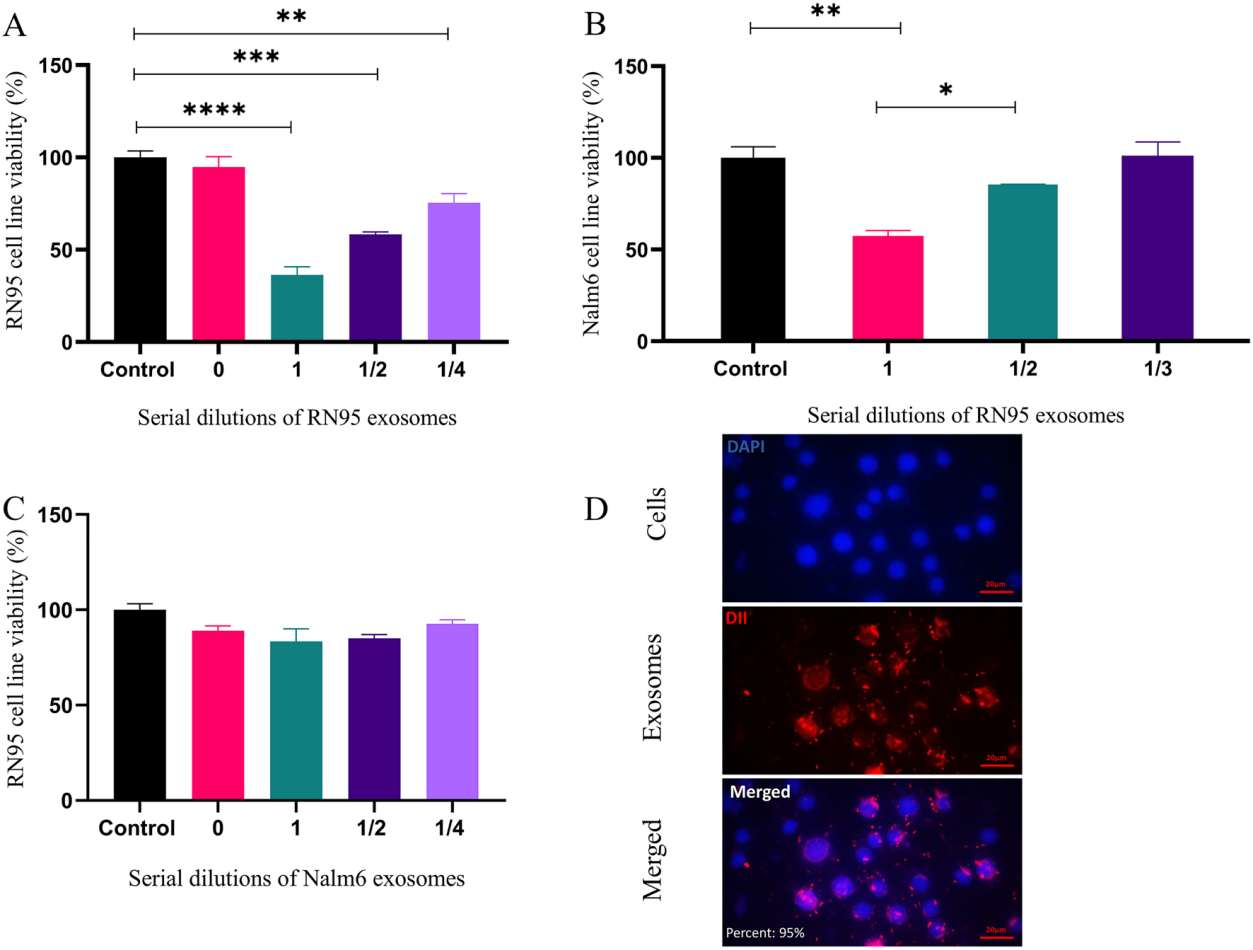
Viability assessment of B-ALL cell lines after treatment with autocrine and exocrine exosomes. (**A**) RN95 and (**B**) Nalm6 cells were seeded into 96-well plates and treated with exosomes extracted from 44 ml RN95 conditioned media. Treated cells were incubated at 37℃ for 48h and cell viability was assessed using MTT assay. (**C**) Nalm6 exosomes with identical concentration to RN95 exosomes in A, were subjected to RN95 cells. Percent viability was not significantly different from that of untreated cells or cells treated with exosome-free media (depicted as 0). Values are mean ± SEM of 3 independent experiments in triplicates. *P < 0.05, **P < 0. 005, ***P < 0.001, ***P < 0.0001. (**D**) Internalization and uptake of exosomes by B-ALL cells. DAPI-labeled B-ALL cells were incubated (24h) with DiI-labeled exosomes (originated from lymphoblastic cells). Related images were captured at the end of the incubation time using fluorescence microscope and a representative example of a triplicate experiment is shown. Scale bar = 20 µm.

Exosomes were collected from 44 ml conditioned media of RN95 and Nalm6 cell lines. RN95 and Nalm6 cells were seeded into 96-well cell culture plates and treated with different dilutions of autocrine exosomes. After 48h incubation, cells′ viability was calculated using MTT assay. Results demonstrated significant dose response reduction in the viability of RN95 cells ]36.31 ± 4.4%, 58.36 ± 1.3% and 75.44 ± 5.03% for 1, 1/2 and 1/4 diluted exosomes, respectively, compared with untreated cells (mean ± SEM, n = 3) [(Fig. 6A). However, no reduced decreased cell viability was shown when RN95 cells were treated with the same volume of Nalm6-derived exosomes (*P* > 0.05) (Fig. 6C). Moreover, Nalm6 cells were treated with different dilutions of exosomes derived from the RN95 cells supernatant for 48h. Nalm6 cells viability was assessed upon MTT assay. Results showed that the RN95 exosomes suppressed Nalm6 cell viability in a dose-dependent manner. However, the reduction in cell viability was less than that of self-exosome treated RN95 cells. (57.45 ± 3.0% vs. 36.31 ± 4.4%, respectively (mean ± SEM, n = 3), *P* < 0.005) (Fig. 6B). In order to investigate the uptake of exosomes by recipient cells, DiI pre-labelled exosomes were added to the RN95 cell line. Cells were then incubated at 4 ℃ for 30 min and stained with DAPI. Fluorescent microscopy confirmed the uptake of exosomes by RN95 cells with a rate of 91.33% ± 3.51 using IMAGE J Software (Fig. 6D).

### The effect of exosomal miR-326 on the viability of B-ALL primary cells

**Figure 7:**
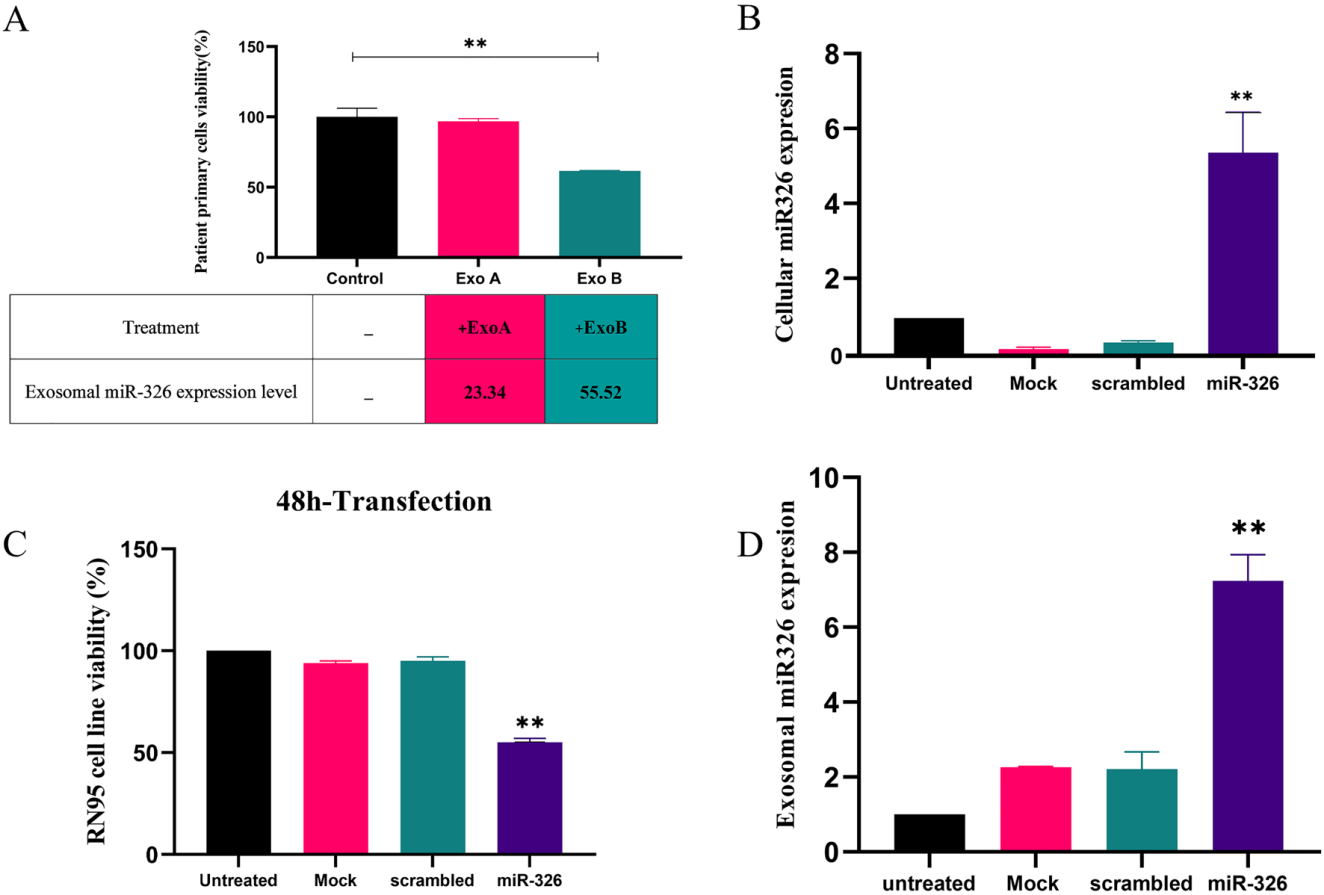
(**A**) The therapeutic effect of exosomal miR-326 on B-ALL patient cells. Patient primary cells were isolated by Ficoll–Hypaque density gradient centrifugation method, then seeded into 96-well plates, and treated for 48h with exosomes derived from patients A or B. Cell viability was assessed using MTT assay. Relative gene expression level of miR-326 was quantified using 2^−ΔΔCt^ method. Regarding the high expression level of miR-326 (55.52) inside the exosomes extracted from patient B, viability of the treated cells was reduced significantly in comparison with the untreated cells and/or those treated with exosomes derived from patient A with lower expression levels of miR-326 (23.34). Exo A, exosomes purified from patient A; Exo B, exosomes derived from patient B; MDR, multidrug resistance. (**B**, **C**) The impact of naked miRNA mimic on RN95 B-ALL cell line. MiR-326 mimic and its related scrambled sequence were delivered into the RN95 cell line using the electroporation method. Following 48h incubation Increased concentration of intracellular miR-326 and cell viability was assessed. Results showed that naked miR-326 mimic alleviates the viability of target cells in comparison with its control groups. (**D**) Increased concentration of miR-326 inside the released exosomes. Followed by 48h post transfection of RN95 cells, exosomes were collected from the cultured media and the expression level of miR-326 was evaluated using RT-qPCR. Values are mean ± SEM; Data are results of 3 independent experiments in triplicates. **P < 0.005, *P < 0.05*.*

Patient mononuclear (MN) primary cells were isolated using lymphodex kit and seeded in a 96-well cell culture plate. Co-culture assay was performed by incubating the seeded cells with the exosomes purified from the plasma patients A and B whose precise exosomal miR-326 concentrations were previously assessed. After 48h incubation at 37℃, MTT assay was performed in order to evaluate the potential therapeutic effect of the exosomal miR-326 on the viability of patient malignant cells. Results showed that the exosomes purified from patient B inhibited the viability of patient primary cells, significantly [61.59 ± 0.2% vs. 100.0 ± 4.3% (mean ± SEM, n = 3), **P < 0.005]. It was proposed that the aforementioned viability reduction was attributed to the increased expression level of miR-326 inside the selected exosomes (ExoB) (2^−ΔΔCt^: 55.52) (Fig. 7A).

### Diminished viability of transfected cells overexpressing ectopic miR-326 accompanied by the sequential cell mediated-ejection of miR-326 into the exosomes

RN95 cells were transfected by miR-326 mimic and its related scrambled sequence using the electroporation method. Following 48h incubation, increased concentration of cytoplasmic miR-326 was confirmed compared with control groups (5.64 ± 0.55 vs. 1.89 ± 0.14 (mean ± SEM, n = 3), *P* < 0.01), respectively. MTT and trypan blue assays showed reduced viability in transfected cells with naked miR-326 mimic compared to their control groups (55 ± 2% vs. 95 ± 2%, (mean ± SEM, n = 3), *P* < 0.01), respectively. Subsequently, exosomes were collected from the media of transfected cells. Increased expression levels of exosomal miR-326 was determined in transfected cells compared with the control groups (7.24 ± 0.55 vs. 2.2 ± 0.01 (mean ± SEM, n = 3), *P* < 0.005), respectively.

## Discussion

Exosomes are currently regarded as non-invasive diagnostic tools that have been utilized in the development of vaccines, as well as drug delivery systems and biomarkers, with potential implications for cancer management. In the present study, exosomes obtained from the plasma of ALL patients and the cell culture media of B-ALL cell lines were effectively isolated with high yield using ultracentrifugation and specific isolation kits. NTA graphs and video captures showed high concentration of exosomes, purified from both techniques, without special pollution, remarkable large vesicles and protein aggregates (Figs. 1A, 2A). Traditional and capillary immunoblotting detected and validated the presence of exosome specific surface markers (Figs. 1B, 2B). Moreover, FE-SEM and FE-TEM images approved more rigorously the spherical and cup shape of the nanoparticles (Figs. 1C, 2C).

Selective packaging of molecules in exosomes and the clinical impact of this phenomenon in cancer has been currently noticed as a heated debate topic in translational medicine^54,55^. It is conceivably hypothesized that lymphoblasts undergoing malignancy use exosomes as vehicles to discard the tumor suppressor miRNA transcripts^56–58^. Bearing this hypothesis in mind, we aimed to examine the presence of a proposed tumor suppressor miRNA, miR-326, inside the isolated B-ALL exosomes and evaluate its impact on leukemia diagnosis and drug resistance.

The rationale for selecting this miRNA was our previous studies confirming the impact of this molecule as a robust cytoplasmic biomarker in ALL^28,34,35^. In the current study, RT-qPCR analysis revealed a significant increase in the expression level of exosomal miR-326 in B-ALL patients compared with the control samples, giving it a diagnostic value for pediatric B-ALL, approved by ROC analysis (Fig. 4). On the other hand, increased concentration of miR-326 was observed in the exosomes isolated from the culture medium of RN95 cells compared with those purified from Nalm6 growth media (Fig. 3). ALL-multidrug resistant patients demonstrated elevated levels of exosomal miR-326 compared with the drug sensitive cases (Fig. 5). These results were consistent with our previous data reporting the cytoplasmic miR-326 as a candidate biomarker with poor prognosis in pediatric B-ALL. Monitoring exosomal-miR-326 during the course of treatment, may help clinicians optimizing therapy decisions. Taken together, the most novel finding to emerge from the abovementioned results is that the exosomal miR-326 can be considered as a promising diagnostic and prognostic biomarker in the context of pediatric B-ALL.

The effect of exosomes on B-ALL cell lines was demonstrated. Results showed that cell viability can be suppressed, dose dependently trough treatment with self and non-self exosomes (Fig. 6A,B). In this context, exosome-mediated autocrine communication between the cells appreared more effective (decreased viability in RN95 cells treated with self exosomes was more intense than that with non-self exosomes). Accordingly, it was previously confirmed that exosomes prefer to target cells having more similarity to their parental origin ^59^. Immunostaining assay illustrated internalization of exosomes in the leukemic cell line followed by its co-culture with resistant RN95 (Fig. 6D), proposing the reason of its mortality. It can be postulated that the drug resistant B-ALL cell line may encapsulate antiproliferative molecules inside the exosomes aiming to conserve tumorigenesis at the intracellular level.

The ingress of vesicles inside the target cells were authenticated using fluorescence microscopy (Fig. 6D). Exosome uptake may occur through endocytosis, receptor ligand interaction and membrane fusion^60^.Furthermore, composition of lipids, integrin and tetraspanins may cause natural targeting of EVs^61^.The abundant exosomal tetraspanins shown in the isolated exosome fractions (Figs. 1B and 2B) might have, therefore, facilitated the exosome uptake.

MicroRNA-326 was identified as a specific component of ALL-derived exosomes fulfilling the role of a tumor suppressor. Identification of miR-326 in all the ALL extracellular vesicle was not conceivable, however, the proportion of exosomes containing miR-326 was significantly enough to show a substantial impact on the viability of target cells. A uniform population of patient malignant primary cells received equal volume of exogenous exosomes with two different miRNA expression levels. Results showed that cell viability was dramatically suppressed in an exosomal miRNA dose dependent manner (Fig. 7A). miR-326 is not asserted to be the only ingredient of the exosomes carrying out the antitumor impact in ALL. However, decreased viability of transfected RN95 cells following the overexpression of miR-326 mimic, confirmed the anti-cancer effect of this specific constituent in B-ALL exosomes (Fig. 7B and C). Moreover, increased expression levels of exogenic miR-326 transported into the released exosomes (Fig. 7D) indicated a potentially defensive behavior of the transfected cells against this tumor suppressor. Incubation of RN95 cell line with Nalm6-derived exosomes induced no-significant cytotoxicity compared to the exosomes derived from RN95 cells which possessed higher concentration of miR-326 (Fig. 6A,C). Altogether, results showed that miR-326 can be considered as one of the exosomal factors which mediates the survival reduction in pediatric B-ALL malignant cells.

To the best of our knowledge, the present study is the first to evaluate the role of exosomal miR-326 in pediatric B-ALL. miR -181a and 181b-5p were previously reported as proliferative exosomal microRNAs^62,63^. Moreover, exosomal miR-146a-5p, miR-181b-5p, and miR-199b-3p were recently introduced as TLR8 agonist ligands facilitating ALL progression^64^. The current findings suggest that exosomes containing miR-326 can help diagnosing cases with native drug resistance as well as facilitating treatment of B-ALL patients. There is abundant room for further progress in determining the mechanism through which miR-326 may suppress the proliferation of tumor cells and detect the involved molecular pathways and target genes. Preclinical studies are also required to understand whether administration of exosomes generated by resistant patients could alleviate symptoms of malignancy in ALL animal models and decrease the tumor burden.

## Supplementary Information

Supplementary Information 1.

Supplementary Information 2.

## Data Availability

Data that support the findings of this study are available from the corresponding author upon reasonable request.
