# Supplementary material for: Plasma-derived exosomal miR-326, a prognostic biomarker and novel candidate for treatment of drug resistant pediatric acute lymphoblastic leukemia
Source: Sci Rep. 2024 Jan 6;14:691. doi: 10.1038/s41598-023-50628-w (PMC12518617; doi:10.1038/s41598-023-50628-w)
Supplement: Supplementary file 1 — Supplementary Information 1. [file 41598_2023_50628_MOESM1_ESM.pdf]

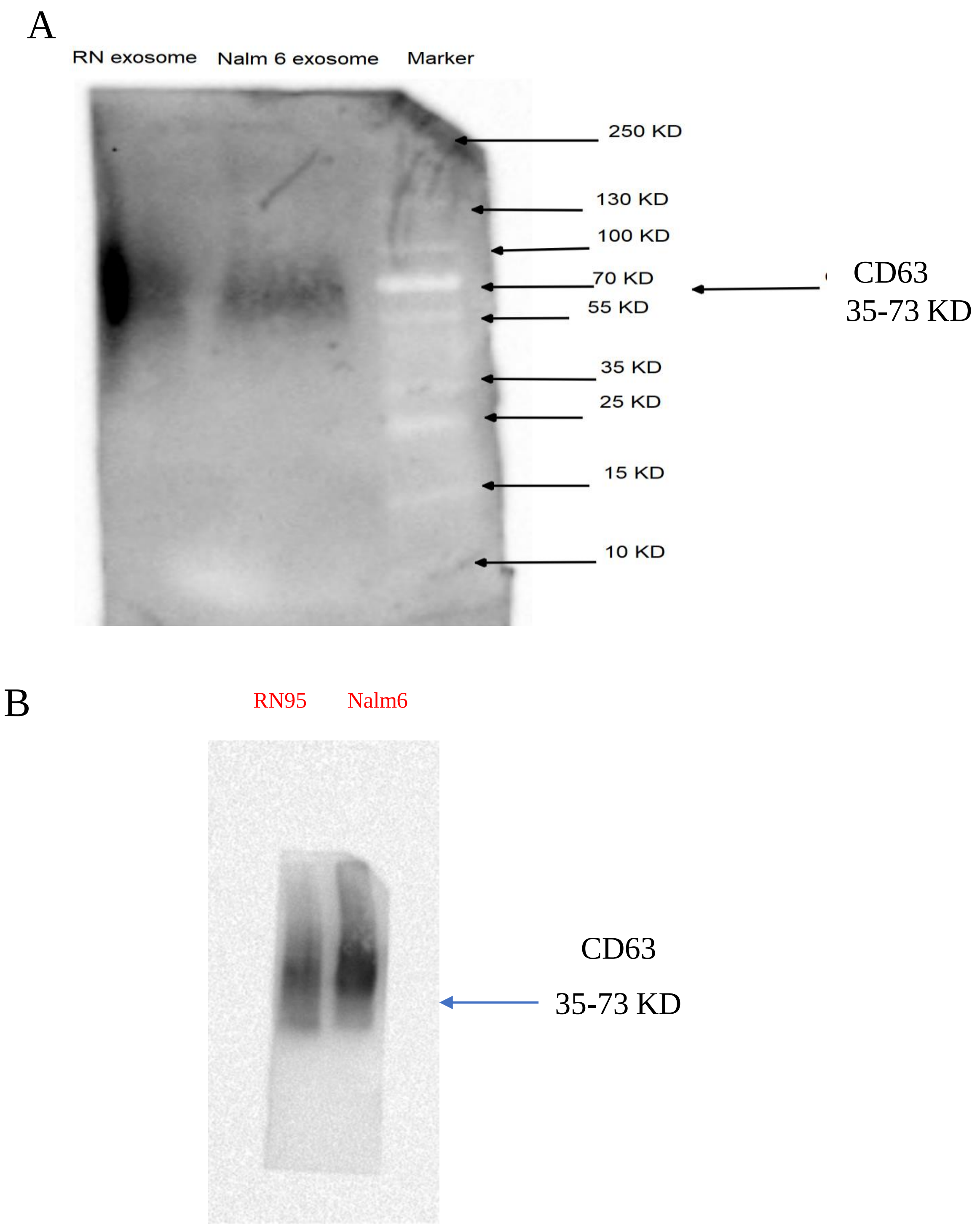

Figure 1B.Original pictures of membrane. CD63 antibody for exosomes (A) and cells (B) from RN & Nalm6 cell lines.

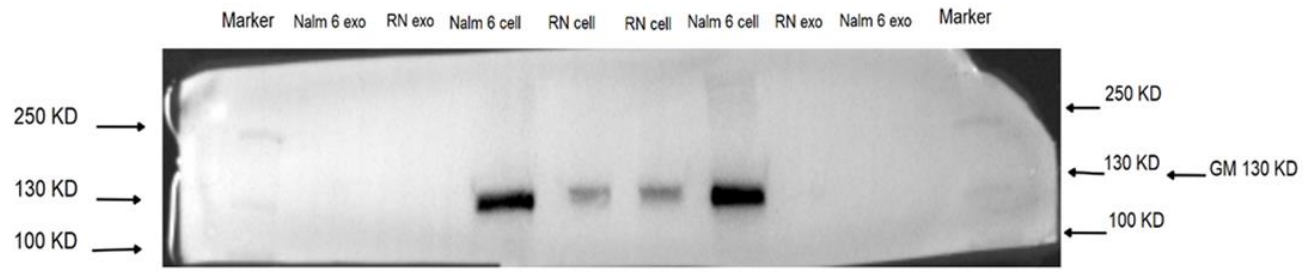

Figure 1B.Original pictures of membrane. GM130 (negative marker) antibody for exosomes and cells from RN & Nalm6 cell lines. Exo = exosome

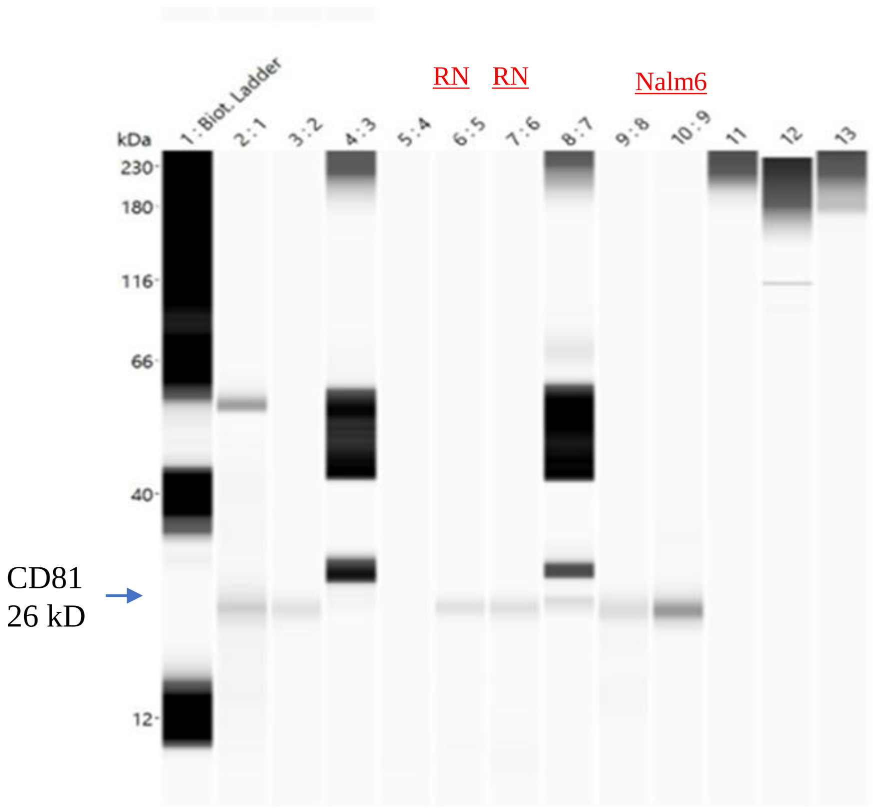

Figure 1B.Original images of Wes simple protein output data. CD81 antibody for exosomes from RN & Nalm6 cell lines.

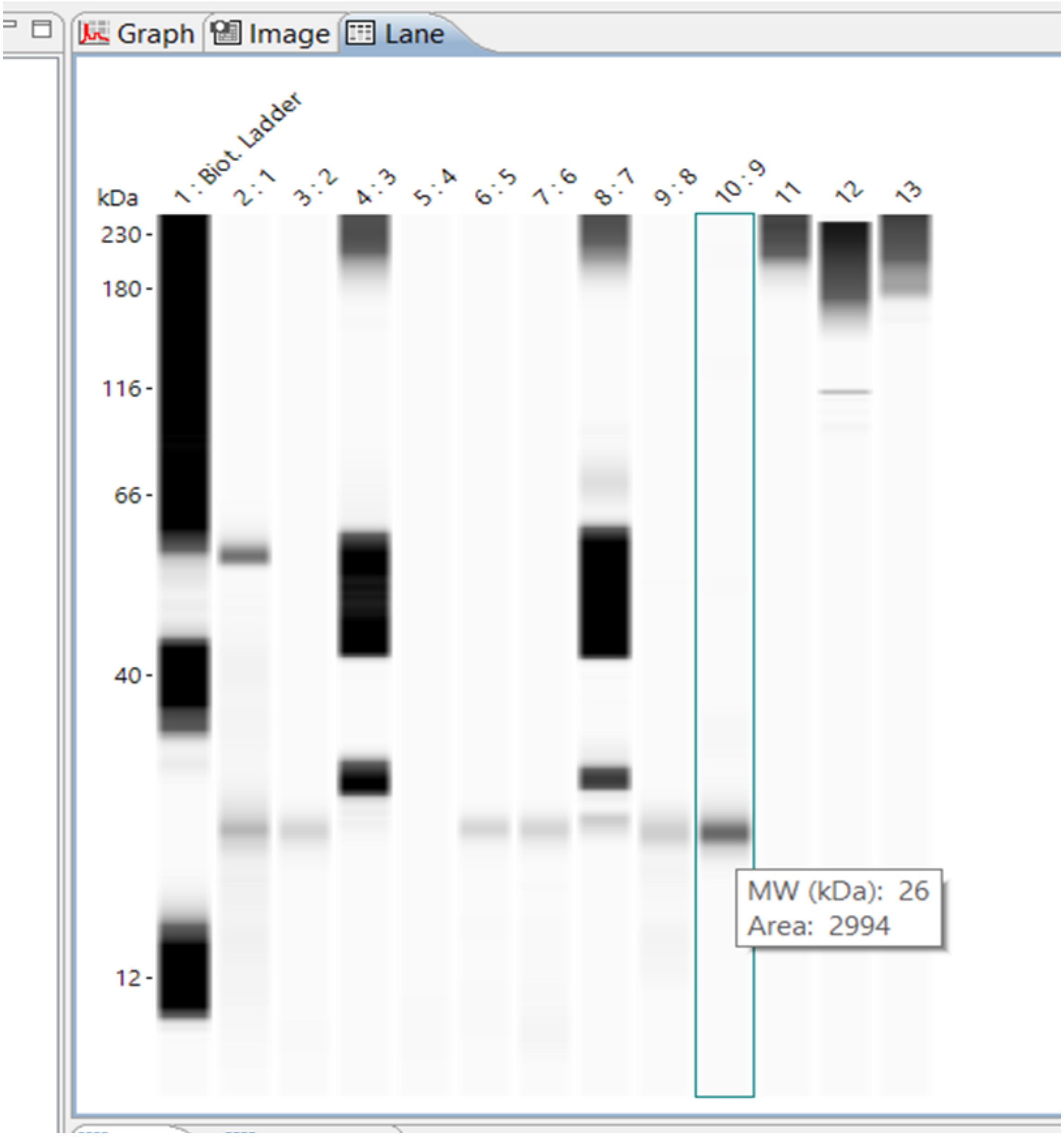

Figure 1B.Original images of Wes simple protein output data. CD81 antibody for exosomes from RN & Nalm6 cell lines.

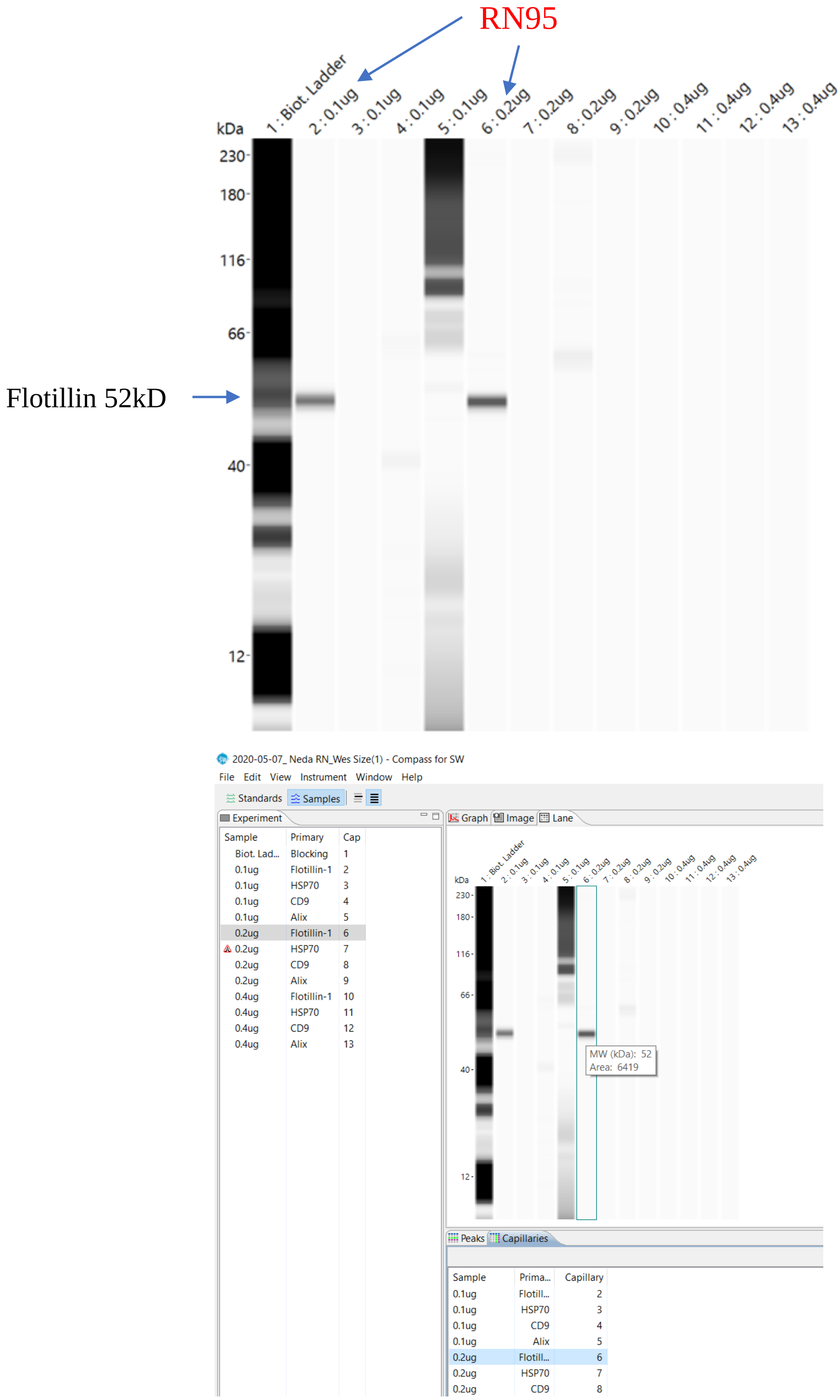

Figure 1B.Original images of Wes simple protein output data. Flotillin antibody for exosomes from RN cell line.

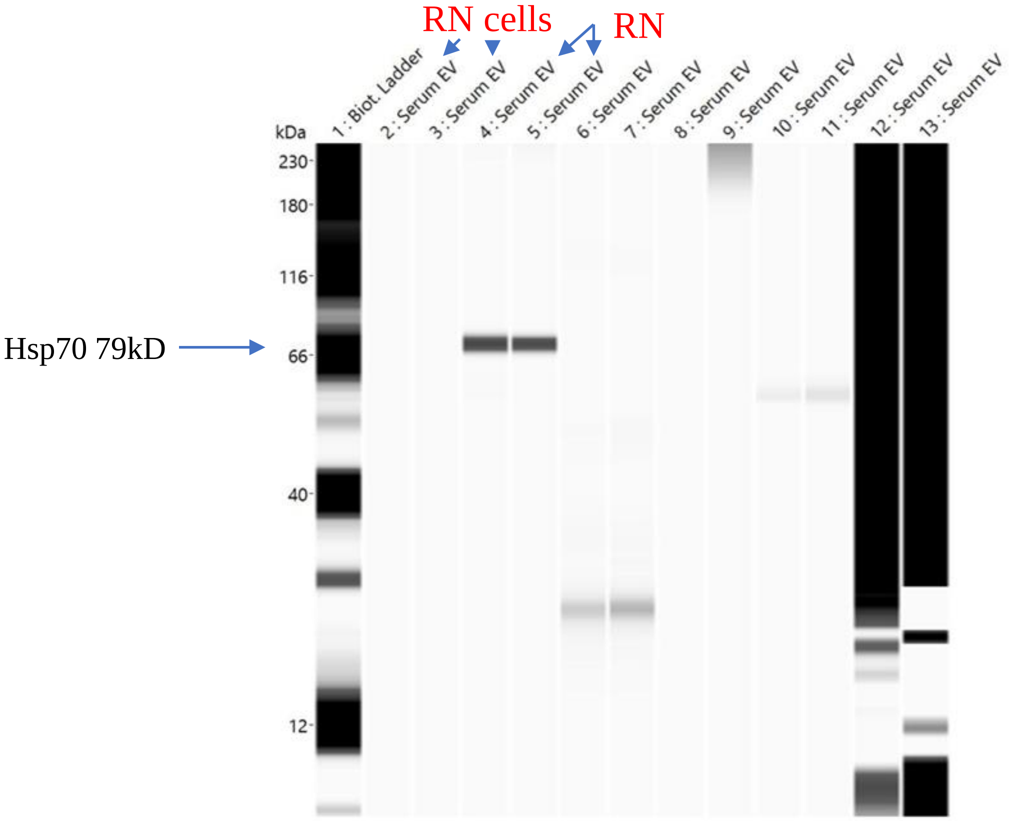

Figure 1B.Original images of Wes simple protein output data. Hsp70 antibody for exosomes from RN cell line.

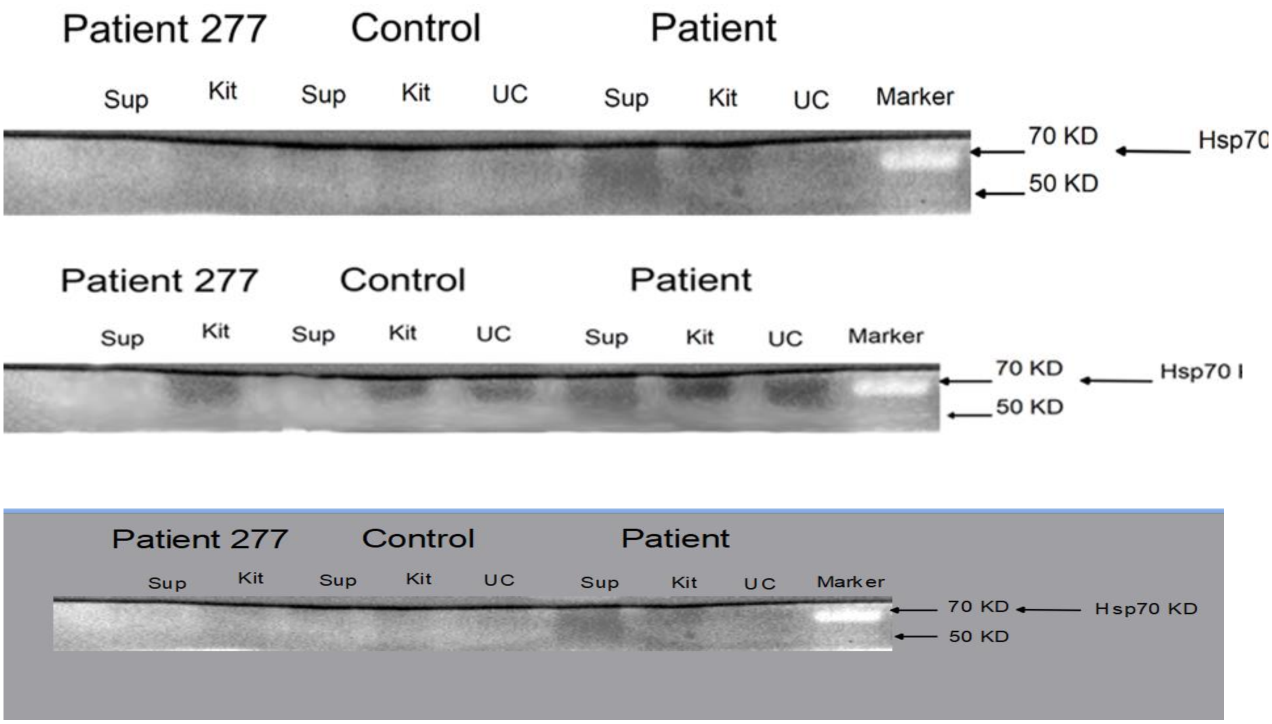

Figure 2B.Original pictures of membrane. <u>Hsp70</u> antibody for exosomes & related supernatant (patient & control).Sup = supernatant; UC = Ultracentrifugation

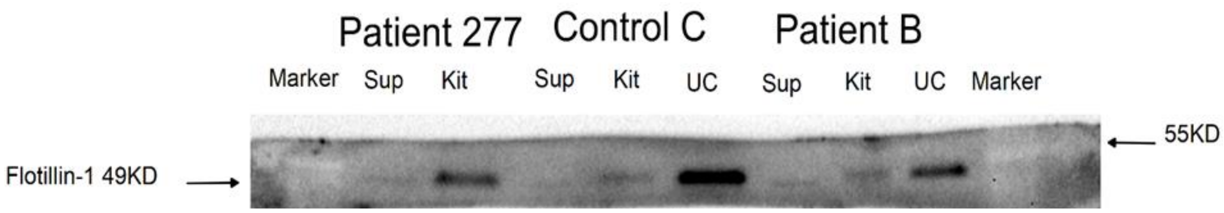

Figure 2B.Original pictures of membrane. <u>Flotillin-1</u> antibody for exosomes & related supernatant (patient & control).Sup =Supernatant; UC = Ultracentrifugation.

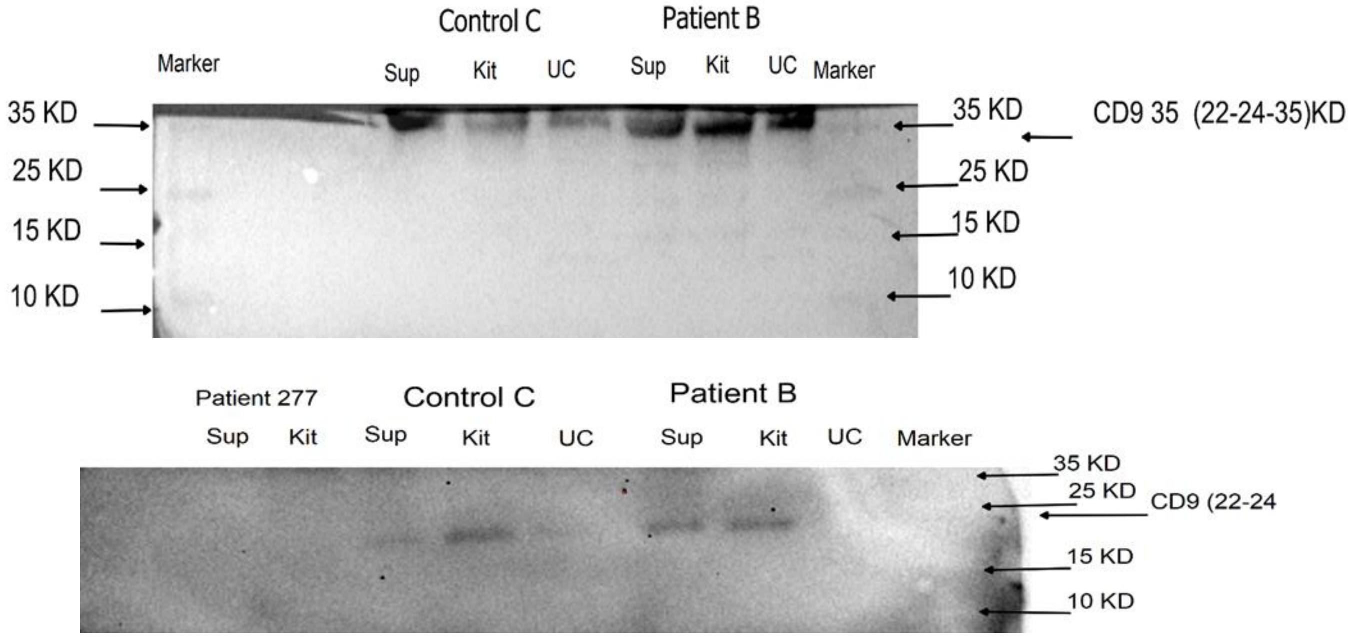

Figure 2B.Original pictures of membrane. CD9 antibody for exosomes & related supernatant (patient & control).Sup = Supernatant; UC = Ultracentrifugation

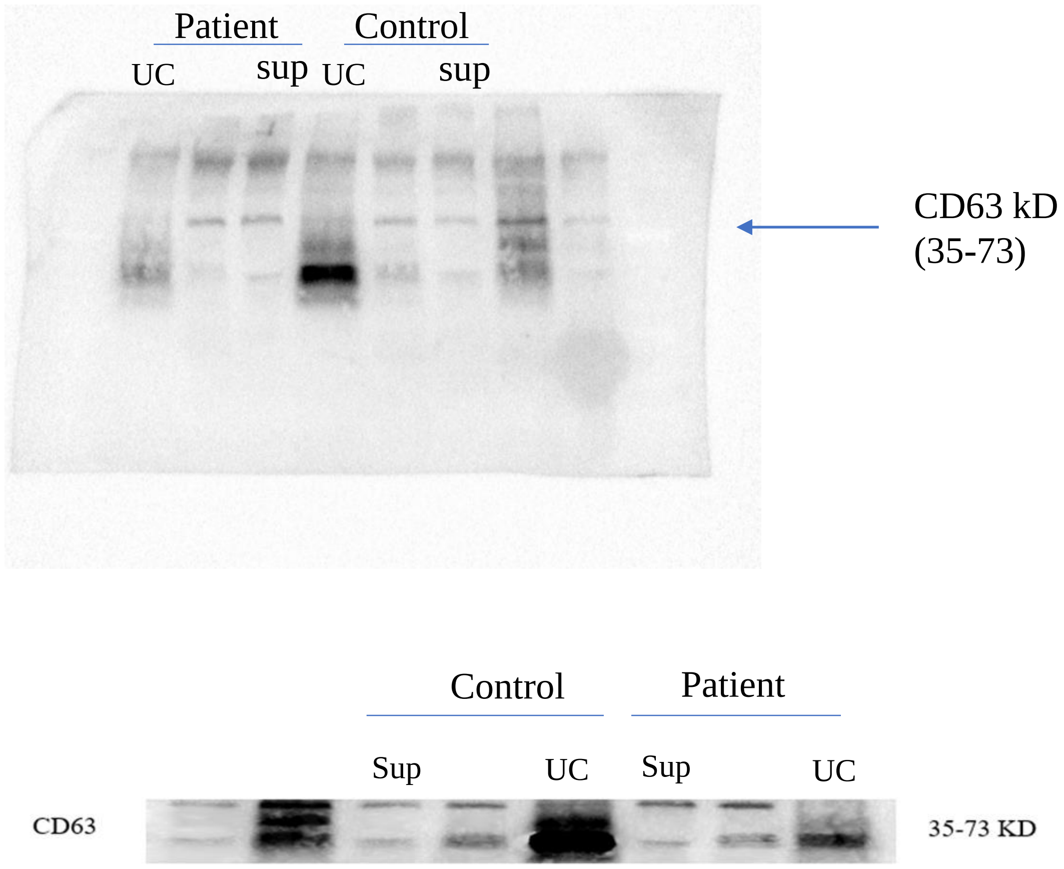

Figure 2B.Original pictures of membranes. CD63 antibody for exosomes & related supernatant (patient & control).Sup = supernatant of exosomes; UC = Ultracentrifugation method

Patient Control

UC sup UC sup

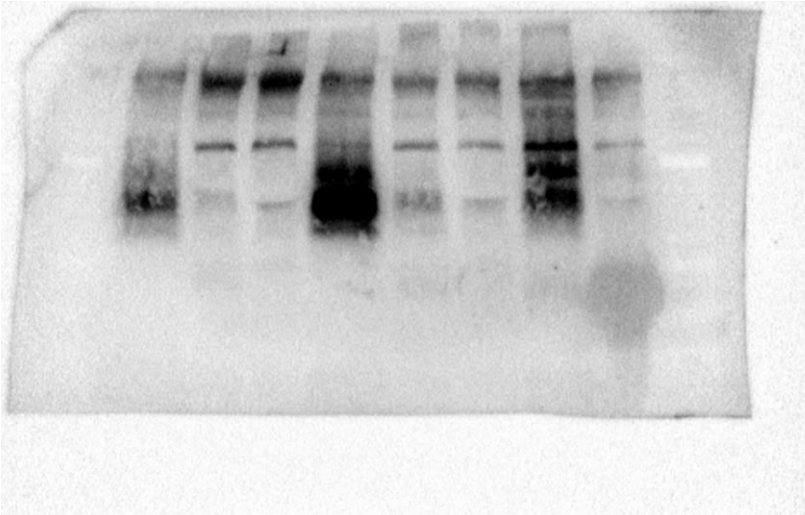

CD63 kD
(35-73)

Figure 2B.Original pictures of membranes. CD63 antibody for exosomes & related supernatant (patient & control). Sup = supernatant of exosomes; UC = Ultracentrifugation method

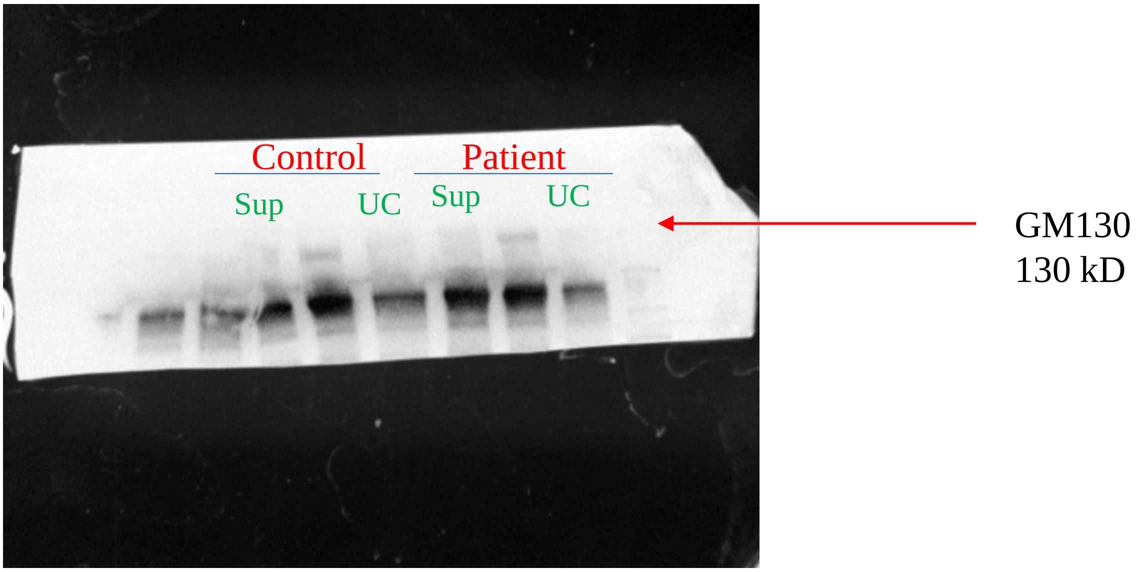

Figure 2B.Original pictures of membranes. GM130(as a negative marker) antibody for exosomes & related supernatant (patient & control). Sup = supernatant of exosomes; UC = Ultracentrifugation method
