# Supplementary material for: Plasma-derived exosomal miR-326, a prognostic biomarker and novel candidate for treatment of drug resistant pediatric acute lymphoblastic leukemia
Source: Sci Rep. 2024 Jan 6;14:691. doi: 10.1038/s41598-023-50628-w (PMC12518617; doi:10.1038/s41598-023-50628-w)
Supplement: Supplementary file 2 — Supplementary Information 2. [file 41598_2023_50628_MOESM2_ESM.docx]

*
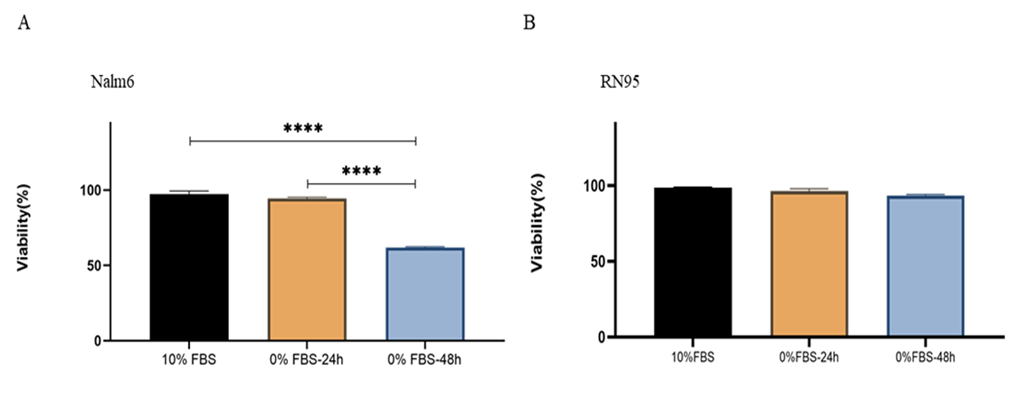
*

*Figure S1.* Effect of FBS- free media on the viability of the B-ALL cell lines, Nalm6 and RN95, for 24h and 48h, respectively, as a time interval needed for exosome harvesting. Nalm6 (A) and RN95 (B) cells were seeded into 96-well plates, then treated with complete and FBS-free media for 24h and 48h, respectively. Cell viability was then assessed using MTT assays. Results showed statistically significant difference between the rates of cell viability after 24h and 48h incubations with FBS deprived culture media for Nalm6. For RN95 cells, results showed no statistically significant difference between the rates of cell viability after 24h and 48h incubations with FBS deprived culture media. Values are mean ± SEM of two separate experiments in triplicates. FBS, Fetal Bovine Serum;**** *P*<0.0001


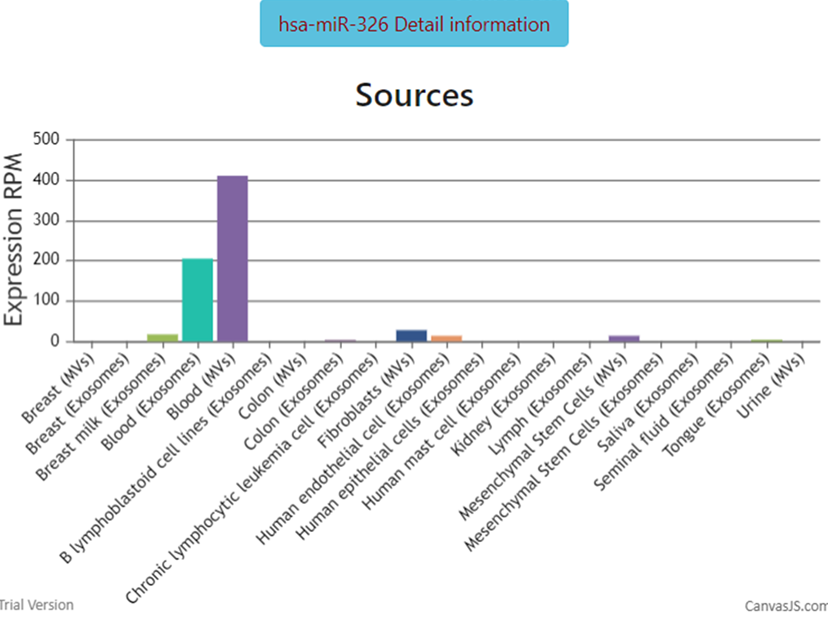


Figure S2. Expression level of miR-326 inside the EVs collected from different human tissues based on EVmiRNA database.
